# Exploring the Perceptions of mHealth Interventions for the Prevention of Common Mental Disorders in University Students in Singapore: Qualitative Study

**DOI:** 10.2196/44542

**Published:** 2023-03-20

**Authors:** Alicia Salamanca-Sanabria, Ahmad Ishqi Jabir, Xiaowen Lin, Aishah Alattas, A Baki Kocaballi, Jimmy Lee, Tobias Kowatsch, Lorainne Tudor Car

**Affiliations:** 1 Future Health Technologies Singapore-ETH Centre Campus for Research Excellence And Technological Enterprise Singapore Singapore; 2 Lee Kong Chian School of Medicine Nanyang Technological University Singapore Singapore; 3 Centre for Health Informatics Australian Institute of Health Innovation Macquarie University Sydney Australia; 4 School of Computer Science University of Technology Sydney Sydney Australia; 5 Research Division Institute of Mental Health Singapore Singapore; 6 Department of Psychosis Institute of Mental Health Singapore Singapore; 7 Institute for Implementation Science in Health Care University of Zurich Zurich Switzerland; 8 School of Medicine University of St. Gallen St. Gallen Switzerland; 9 Centre for Digital Health Interventions Department of Management, Technology, and Economics ETH Zurich Zurich Switzerland; 10 Department of Primary Care and Public Health School of Public Health Imperial College London London United Kingdom

**Keywords:** interventions, students, mobile health, mHealth, mental health, mental disorders, university, common mental disorders, anxiety, depression

## Abstract

**Background:**

Mental health interventions delivered through mobile health (mHealth) technologies can increase the access to mental health services, especially among university students. The development of mHealth intervention is complex and needs to be context sensitive. There is currently limited evidence on the perceptions, needs, and barriers related to these interventions in the Southeast Asian context.

**Objective:**

This qualitative study aimed to explore the perception of university students and mental health supporters in Singapore about mental health services, campaigns, and mHealth interventions with a focus on conversational agent interventions for the prevention of common mental disorders such as anxiety and depression.

**Methods:**

We conducted 6 web-based focus group discussions with 30 university students and one-to-one web-based interviews with 11 mental health supporters consisting of faculty members tasked with student pastoral care, a mental health first aider, counselors, psychologists, a clinical psychologist, and a psychiatrist. The qualitative analysis followed a reflexive thematic analysis framework.

**Results:**

The following 6 main themes were identified: a healthy lifestyle as students, access to mental health services, the role of mental health promotion campaigns, preferred mHealth engagement features, factors that influence the adoption of mHealth interventions, and cultural relevance of mHealth interventions. The interpretation of our findings shows that students were reluctant to use mental health services because of the fear of stigma and a possible lack of confidentiality.

**Conclusions:**

Study participants viewed mHealth interventions for mental health as part of a blended intervention. They also felt that future mental health mHealth interventions should be more personalized and capable of managing adverse events such as suicidal ideation.

## Introduction

Common mental disorders (CMDs) such as depression and anxiety are among the leading causes of worldwide health-related burdens [1]. This has been further exacerbated by the COVID-19 pandemic, which has increased the prevalence of depression by an estimated 25% worldwide [2]. In Singapore, major depressive disorder has a lifetime prevalence of 6.3%, whereas generalized anxiety disorders have a lifetime prevalence of 4.8% of the population [3]. The median age of onset of CMDs in Singapore is 22 years and most people with CMDs do not seek treatment within the first year of onset [4]. Furthermore, only 32% of those who have ever been diagnosed with a CMD have sought help from a specialized service provider [5], with a notable treatment gap identified among youth [6].

The transition to university coincides with a critical period characterized by psychosocial stressors (eg, separation from family and pressures associated with academic work), in conjunction with sleep disruption and unhealthy lifestyle habits (eg, sedentarism). Most mental disorders develop in early adulthood and are associated with a major delay in seeking treatment [7]. In addition to this, the transition to university coincides with a high-risk period for maladaptive coping, CMDs, unhealthy lifestyle habits, and academic failure, which suggests that prevention and timely intervention are important during this period. This is coupled with limited access to evidence-based interventions because of various factors including the fear of being stigmatized, a low perceived need for mental health support, poor mental health literacy, and limited access to mental health professionals [8,9].

Mobile health (mHealth) interventions could address some of the existing barriers to mental health treatment by being scalable, more accessible, private, and personalized. These interventions promote self-care, which is defined as specific actions taken to promote and maintain health including emotional, mental, and physical health without the assistance of a health care worker [10]. Research shows that mHealth interventions have the potential to improve mental health outcomes among youth [11] who may be more open to using these interventions [12]. For example, a systematic review of randomized controlled trials of mHealth intervention for adolescents and young adults showed that the interventions significantly improved symptoms in multiple outcomes, including depression, anxiety, and stress [13]. Previous studies in Singapore have shown that young adults had positive attitudes toward mHealth interventions, although the use of these interventions was low [14,15]. This was attributed to a lack of willingness to pay and the preference for apps in their native languages, such as Mandarin [15].

mHealth interventions are diverse and include SMS text messaging to more recent and innovative approaches such as conversational agents (CAs). CAs are computer algorithms designed to simulate human conversation textually or by speech through an interactive interface [16]. They are increasingly used in mental health and have been shown to improve depression symptoms [17]. The ability of CA-delivered intervention to establish therapeutic alliances with users [18] makes it attractive as an intervention modality to increase user engagement and adherence to mHealth intervention [19].

Despite increasing evidence supporting it, mHealth interventions typically reported high dropouts and low uptakes among users [20]. Research suggests that highly personalized intervention could improve user adherence through specific feedback, suggestions, and content that are tailored to the users’ needs [20,21]. Interventions that included end users in the design process also tend to be more effective compared with those designed based on the existing system [22]. This suggests the importance of engaging relevant stakeholders in the development of an mHealth intervention to better tailor the intervention to their needs and preferences.

The development of mHealth is complex, context specific, and requires inputs from various relevant stakeholders [23]. A qualitative study on the development of health promotion CA has shown that youth have different preferences for digital interventions compared with those of adults [24]. Recent studies have also shown the complex needs and preferences in digital intervention from both patients and carers including counselors and parents [25,26]. These studies, however, are context specific and might not be generalized to our context in Singapore [27]. Furthermore, no studies have looked at Singaporean youth’s needs and acceptability of mHealth intervention that is sensitive to their cultural identity.

Singapore is a densely populated city-state in Southeast Asia with approximately 5.4 million people, comprising Chinese, Malay, and Indian populations and smaller proportions of other ethnicities [28]. A multiethnic country such as Singapore faces additional challenges as this cultural diversity leads to varying perspectives on mental health [29]. Findings based on other contexts might result in different responses without cultural sensitivity to the local context [30]. For example, studies have shown that the Singaporean Chinese communities view mental health as a shameful condition that will lead to a loss of social standing [29,31]. A cross-sectional study also showed a greater desire for social distance among young Chinese people toward people with mental illness compared with young Singaporeans of other ethnicities [31]. To better understand the local context, this study aimed to lay the groundwork for the development of mHealth interventions for the prevention of CMD among university students in Singapore. We aimed to further contextualize students’ experiences within the support provided by mental health supporters such as faculty members tasked with student pastoral care and mental health professionals treating students. This study has the following objectives:

1. Identify barriers and facilitators related to mental health care services and mHealth interventions for university students.
2. Identify the perception, needs, and preferences of university students and mental health supporters on self-care models and mHealth interventions.
3. Explore the cultural relevance of the development of mHealth interventions in Singapore.

## Methods

### Study Design

This study comprised 6 focus group discussions (FGDs) with university students and 11 interviews with mental health supporters via the videoconferencing platform, Zoom (Zoom Video Communications, Inc). The advertisements for the study were distributed via a social media group for students at a university in Singapore. For the interviews, email invitations to close contacts of the research team and snowball sampling were used. All participants gave their informed consent.

### Ethics Approval

Ethical approval was obtained from the Institutional Review Board of Nanyang Technological University (number IRB-2018-11-03).

### Data Collection

In the FGDs, we included (1) full-time undergraduate students at a local university (aged 21-34 years); (2) those who spoke English; and (3) those who did not have psychosis symptoms, as the planned intervention was not designed to address the needs of users with psychosis symptoms. Participants in the study were not excluded because of the severity of their depression or anxiety symptoms. After the FGDs, the research team offered the participants information about different helplines in Singapore, as well as mental health services at the university. Eligible participants completed a recruitment survey that included their sociodemographic information, confidence in using digital technology, and self-reported symptoms of depression and anxiety collected as demographic information and not intended for a formal diagnosis (Multimedia Appendix 1). We undertook 6 FGDs with an average of 5 participants. The FGDs were conducted by 2 local facilitators who were independent of the research team with no personal interest in the research topic. Two researchers (AIJ and XL) scribed for the sessions and took field notes. Two researchers (AS and AA) attended all the FGD sessions as observers and did not make contributions to the discussion. The FGDs lasted on average 90 minutes following the discussion guide (Multimedia Appendix 2). The guide includes a brief description of the proposed mHealth intervention to elicit responses from participants (Multimedia Appendix 2).

We included mental health supporters, specifically referring to individuals with an interest in the mental health of university students, such as faculty members tasked with student pastoral care and mental health professionals including counselors, psychologists, a clinical psychologist, a psychiatrist, and a mental health first aider with at least 1 year of experience working with university students with CMDs. Eligible participants completed a short survey on their sociodemographic information, perception of CMDs in university students in Singapore, and perception of mHealth interventions (Multimedia Appendix 3). The sampling was carried out until saturation (no new themes identified) based on an iterative analysis of the field notes and agreement among the researchers [32]. The interviews were conducted by 3 researchers (AS, AA, and AIJ). One researcher (XL) took the field notes for all the interviews. The interviews lasted around 45 to 60 minutes following the interview guide (Multimedia Appendix 4).

### Data Analysis

We reported this study in line with the Consolidated Criteria for Reporting Qualitative Research (Multimedia Appendix 5). The audio recordings of the FGDs and interviews were transcribed verbatim by 2 researchers (AIJ and XL) and analyzed using thematic analysis on ATLAS.ti version 9. The analysis followed the six-step process of Braun and Clarke [33,34], using an interpretive approach through iterative phases of (1) data familiarization, (2) code generation, (3) theme development, (4) review of candidate themes, (5) theme refinement, and (6) writing up. Table 1 describes the phases in detail.

**Table 1:** Thematic analysis phases following Braun and Clarke’s [33,34] 6-step process.

Phase	Description
Data familiarization	FGDs^a^ and interviews were initially transcribed automatically using the Zoom platform functions. Two members of the research team (AIJ and XL) reviewed the transcripts and recordings for errors. Additional corrections to transcripts were reviewed by a third researcher (AA). Comments from participants that had been written in the meeting chat were incorporated into the analysis after reviewing the context. XL and AIJ engaged with the focus group and interview transcripts by reading and noting down initial thoughts and observations. After familiarization and with a revision of a third researcher AS, XL, and AIJ led the subsequent steps of the data analysis process, with the rest of the team members acting as “critical friends” involved in reviewing candidate codes and themes and offering points for reflection and alternative explanations.
Code generation	XL and AIJ generated initial codes independently and engaged in discussions regarding the codes for the entire sample of FGDs and interview transcripts. In thematic analysis, codes are short labels that represent important features of the data relevant to answering the research questions; for example, comments such as “pair the app with human professional,” “have a feature like a platform for you to post your feelings etc, then experts/other users can get back to you,” or “I mean for mental health apps right, I wish to see a feature where you can actually speak to the health care professionals” were grouped under the code “preference for expert support in app.” These initial codes were collated, mapped for similarities, and discussed as a group in weekly meetings.
Theme development	The final list of codes generated by XL and AIJ was reviewed by 3 other members of the research team (AS, AA, and LTC), who made suggestions to merge or separate some codes. XL and AIJ used the team inputs to implement further changes to the list of codes and then grouped the codes together into a tentative set of overarching themes and subthemes. For the purposes of this study, themes are understood as a collection of similar codes that provides detail about (1) the participants’ views on CMDs^b^ and healthy lifestyle and (2) digital health solutions.
Reviewing themes	The final list of themes and subthemes was reviewed by AS, AA, and LTC to examine whether they told a convincing story of the data (one that answered the research questions). Raw data were revisited under each theme and subtheme to ensure coherence and consistency. The whole team discussed and came to a consensus on the revisions to the final list of themes (eg, renaming codes or rearranging some of the subthemes).
Defining and naming themes	Names and descriptions for each theme were written by XL and AIJ and discussed with the rest of the team.
Producing the report	Finally, all authors were involved in writing the analysis and findings. AIJ and XL wrote the initial report, and it was discussed and reviewed by the team.

^a^FGD: focus group discussion.

^b^CMD: common mental disorder.

Three researchers (AIJ, AS, and LTC) had previous training and experience with qualitative research. The final set of themes was based on iterative discussions and refinement to examine the coherence of the codes within the themes and the validity of the themes to the data set. All authors collectively agreed on the formulation of the themes. Participants only took part in the FGDs and interviews and were not asked to comment on the findings. The quotations supporting the themes and subthemes are provided in Multimedia Appendix 6.

## Results

### Participants

A total of 30 full-time undergraduate students participated in the FGDs (Table 2). The mean age was 22.74 (SD 1.36) years, and most were Chinese (27/30, 90%). Most of the students studied Arts and Social Sciences (14/30, 47%) and Mathematics and Engineering (9/30, 30%) majors. Half of the students reported having had symptoms of anxiety (15/30, 50%) and depression (15/30, 50%) for several days. Finally, most (23/30, 77%) reported high confidence in their use of digital technology.

A total of 11 mental health supporters participated in the one-to-one interview (Table 3). Most of them (7/11, 63%) had >5 years of experience working with university students with CMDs. Most were counselors (3/11, 27%) and faculty members (3/11, 27%) with at least a master’s degree (8/11, 73%); 2 (18%) were clinically trained professionals, namely a clinical psychologist and a psychiatrist. They also reported confidence in their use of digital technology (9/11, 82%) and found technology to be helpful in their daily life (10/11, 91%).

**Table 2:** Characteristics of the university students (n=30).

Variables		Values
**Sex, n (%)**		
	Female	19 (63)
	Male	11 (37)
Age (years), mean (SD; range)		22.95 (1.89; 21-35)
**Ethnicity, n (%)**		
	Chinese	27 (90)
	Malay	2 (7)
	Indian	1 (3)
**University major**, **n (%)**		
	Arts and Social Sciences	14 (47)
	Mathematics and Engineering	9 (30)
	Science	4 (13)
	Accountancy and business	2 (7)
	Health sciences	1 (3)
**Depressive symptoms, mean (SD)**		1.96 (0.77)
	Not at all, n (%)	9 (30)
	Several days, n (%)	15 (50)
	More than half the days, n (%)	4 (13)
	Nearly every day, n (%)	2 (7)
**Anxiety symptoms, mean (SD)**		1.93 (0.86)
	Not at all, n (%)	9 (30)
	Several days, n (%)	15 (50)
	More than half the days, n (%)	4 (13)
	Nearly every day, n (%)	2 (7)
**IT confidence, mean (SD)**		4.82 (0.39)
	Very confident, n (%)	23 (77)
	Confident, n (%)	7 (23)

**Table 3:** Characteristics of mental health supporters (n=11).

Variables		Values
**Sex, n (%)**		
	Female	6 (54)
	Male	5 (45)
**Age (years), n (%)**		
	<25-45	8 (72)
	>45	3 (27)
**Working experience (years), n (%)**		
	<1-2	2 (18)
	3-5	2 (18)
	6-10	2 (18)
	>10	5 (45)
**Profession, n (%)**		
	Clinical Psychologist	1 (9)
	Counselor	3 (27)
	Faculty members	3 (27)
	Psychiatrist	1 (9)
	Others	3 (27)
**Academic degree**, **n (%)**		
	Bachelor’s degree	2 (18)
	Master’s degree	8 (72)
	Doctoral degree	1 (10)
**Technological competence, mean (SD)**		5.18 (0.87)
	Competent, n (%)	9 (82)
	Average, n (%)	2 (18)
**How helpful do you find technology to be in your everyday life? Mean (SD)**		5.27 (0.91)
	Very helpful, n (%)	0 (0)
	Helpful, n (%)	10 (91)
	Average, n (%)	0 (0)
	Mildly helpful, n (%)	1 (9)

### Findings

The analysis of FGDs and interviews surfaced 3 themes related to perception, preferences, and challenges to healthy lifestyle, mental health services, and mental health campaigns for university students. We identified 2 main themes on the factors related to the adoption of mHealth interventions in general and specifically to the proposed CA intervention. Finally, we also identified 1 theme related to the cultural relevance of the design and development. Multimedia Appendix 6 describes the most relevant quotations related to each theme and subtheme.

### Healthy Lifestyle as Students

This theme revolves around the intersection between the perception of a healthy lifestyle and participants’ personal life. For university students, a healthy lifestyle includes a sense of balance between the ideal state of health and their responsibilities as students. The following 3 values were evident: (1) the importance of having a “balanced lifestyle,” (2) the importance of having a “healthy daily routine,” and (3) the importance of mental health. Most of the students mentioned physical health, diet, and restful sleep, individually or concerning having a “healthy lifestyle” and a “balanced lifestyle.” This idea of a “balanced lifestyle” may also include other aspects of healthy living beyond physical health. For instance, 1 student mentioned as follows:

I think that a healthy lifestyle will be across all the aspects of health...so like, physical, social, emotional, [and] cognitive health.

FGD1, Student 001

A “healthy routine,” according to the students, involved having a reasonable sleep schedule while balancing their university work and social life. For instance, 1 student mentioned:

I don’t think [it is just about] sleeping early and waking up early, it could also be like sleeping late and waking up late, but having a consistent timing for sleep, as long as like it’s okay with your school or work.

FGD6, Student 027

Some also expressed the importance of mental health and mentioned the importance of being aware of their actions and thoughts and maintaining mental wellness.

The students shared that their current circumstances prevented them from engaging in their preferred activity toward a healthy lifestyle. Some barriers included the effect of COVID-19 on maintaining a healthy social lifestyle. Others included the lack of motivation and feeling hampered by bad habits such as procrastination and bad eating habits. Most cited university commitments as barriers to engaging in healthy lifestyle behaviors. Overall, students perceived a healthy lifestyle as a delicate balancing act that is affected by their responsibilities as students and external circumstances, including the pandemic restrictions. A student for instance mentioned that:

...sometimes I have obligations to fulfil. For example, I have work to do, but at the same time I wanted I go out with my friends,...so I end up losing sleep or my mealtimes become irregular.

FGD1, Student 001

### Access to Mental Health Services

This theme is related to perception, barriers, and facilitators of accessing professional mental health services in Singapore as students. The following subthemes emerged: (1) access to friends and family, (2) stigma surrounding mental health issues, and (3) trust in mental health services.

The students cited at least one mental health service either available in public or at their university. Importantly, many students felt constrained to approach professional services because of the cost and lengthy processes involved such as the long waiting time. However, we noted a strong preference for them to seek help from friends and family or self-help through web-based resources and apps rather than seek professional services. Students felt that family and friends could provide immediate support to them in times of need. Students also felt that web-based resources such as social media and websites are freely available and more accessible:

I [wrote] friends because they [are at the] same point [of time] in my life. So, my experience will be kind of like theirs. So maybe they kind of understand me better. Then I just maybe seek their opinions. But if the problem persists, then I might go to my family.

FGD1, Student 016

I think [I] would also look at online resources such as YouTube videos or online forums to see how...others react to [my mental health issues]? And perhaps go to specific online communities that are geared towards mental health to talk to people.

FGD6, Student 018

Fear of social stigma, especially the sense of being judged by others, pushed students into seeking help primarily with their close contacts rather than reaching out to someone new. However, students also mentioned that they felt less stigmatized among their peers with whom they were more open to sharing their concerns. This was related to their level of trust in their friends or family members compared with that of professional services:

For me, I’ll just most likely talk to friends who I’ve already established a connection with, and like we’ve already talked about mental health issues before, because I feel that I’ll receive less stigma than trying to talk to someone new.

FGD3, Student 009

Other common concerns include privacy and confidentiality issues related to the sharing of personal information with parents or potential employers. This was perceived as a breach of trust for students and could prevent them from seeking professional services. However, we noted that these concerns were not shared by students with direct experience of the services. Furthermore, despite these concerns, some students still consider seeking help from mental health professionals when their issues became too severe for their immediate support network and professional intervention may be the more appropriate course of action. For example, a student mentioned:

Most of the time I will approach my friends first, because like I hang out with them more, they probably know me better in that sense or they can relate to the problems I faced. Then, maybe next will go to my—my siblings...when both friends and family can’t help, then I might go to [the] counsellor.

FGD3, Student 032

### Roles of Mental-Health Promotion Campaigns

Students were generally aware of the mental health campaigns and mentioned campaigns such as “Beyond the Label” by the National Council of Social Services or those run by local organizations such as Silver Ribbon and CHAT. Those who were unaware of publicly organized campaigns mentioned the campaigns run by their university.

Those who were more aware of these campaigns shared that the campaigns were able to generate conversations among students about mental health issues. Students acknowledged that the campaigns and services ran by public organizations and various health institutions were useful in providing access to mental health resources and existing helplines. This was perceived as a positive start toward improving mental health by disproving the “stigma of those with mental health issues” (FGD6: Student 018). Some also acknowledged the role of the pandemic in foregrounding the importance of mental health with much more participation among the public to share their perspectives in public spaces. For example, 1 student mentioned as follows:

...because of the pandemic situation, more of, more of like, I guess, more in terms of the [online] community has uh spread—helped spread or market this programme, so I guess that helps.

FGD6, Student 018

Students also shared that the campaigns were too short, insufficient, irrelevant, unenticing, or did not have enough reach to be useful for everyone. For instance, although these campaigns were able to increase the awareness of the available resources, participants felt it might still be difficult for individuals to reach out to mental health services or use resources promoted by these campaigns. Some also felt that these campaigns, especially those on social media, were mainly targeting youth and might not address the wider community.

### Preferred mHealth Engagement Features

This theme encompasses the views on the design elements of mHealth interventions that can sustain user engagement. The following 3 subthemes were identified based on the perceptions of both students and mental health supporters on the mental health app they were currently using: (1) basic app features, (2) active engagement features, and (3) self-awareness features. Participants cited at least 1 known mental health app they used or had heard of during the FGDs and interviews, such as *Wysa,* *Woebot,* and *Headspace*.

Regarding basic features of the mHealth intervention, students and mental health supporters mentioned the following preferences for mental health apps: (1) personalizing the app to suit individual needs; (2) making the app available at low or no cost; (3) having an aesthetically pleasing design interface; (4) ensuring that the app is backed by research and endorsement from trusted parties, such as the government, social media, or friends; and (5) ensuring that the app has a user-friendly and intuitive design (easy to use).

Regarding active engagement features, students mentioned the following features that would increase user engagement: (1) using gamified activities, (2) providing monetary rewards to motivate the use of the app, (3) using reminders and prompts to encourage accountability and action, and (4) story-based narratives within the app. There were suggestions for specific content, such as providing mindfulness exercises and guided activities, by both students and mental health supporters. However, many expressed that the content in existing apps tends to be locked behind paywalls and is typically not available to them.

Mental health supporters suggested content related to cognitive behavior therapy because of its well-defined framework and the inclusion of homework components that can be easily delivered and completed by users. Students and mental health supporters highlighted the importance of social features in interacting, comparing, and competing with friends and other users of the app. Importantly, both students and mental health supporters preferred that the app included real human support remotely or use a blended approach to use the app between in-person sessions. For example, a student mentioned:

I wish to see a feature where you can speak to the health care professionals, so to people, people who are at the back and the other side. Not just bots.

FGD5, Student 044

Participants also suggested the inclusion of features that could provide psychoeducation and information about one’s mental well-being, increase self-awareness, and assist them with keeping track of personal mental health information. Features such as behavior tracking, mood tracking, and journaling functions were shared by both students and mental health supporters as ways of self-monitoring despite concerns about manual data entry. Another suggestion was to have informative articles on relevant health topics such as healthy diets and psychoeducation content personalized to their interest.

### Factors Influencing the Adoption of mHealth Interventions

A total of 3 subthemes emerged during the discussion on future mHealth interventions for mental health and well-being with a focus on CA-delivered intervention. These included possible functions of the app, the “humanity” of the CA, and perceived technological limitations of the CA.

Most mental health supporters viewed the proposed mHealth intervention as complementary to the services and support they provided. This could be in the form of CA-guided exercises that clients could do on their own between therapy sessions via a blended approach supported by the therapist during sessions. The mHealth intervention could also function as a triaging service before the first counseling session. For example, a mental health supporter mentioned:

[This] could be actively promoted...to the patient when they are waiting for their consultation in restructured hospitals so that they know that [this] service exists...and they could be before they see their specialist.

Mental health supporter 022

Some students and mental health supporters also see the potential of the proposed app as a prevention tool through symptom monitoring or for crisis management, especially when professional help is not immediately available.

Students mostly expressed concerns regarding the use of the CA for mental health, specifically related to the humanness of the CA. Many perceived CA to be cold and unemotional with static and unnatural responses. They also mentioned how they preferred to have a “human touch” in the responses and a person who can better relate to the issues presented. We note that most students and mental health supporters expect CA to function as humans or therapists, which might suggest their insistence that CA will not replace human support. Some, however, also acknowledged other auxiliary functions of the CA that may complement existing services as mentioned previously.

Students and mental health supporters mentioned the perceived technological limitations of CA-based interventions specifically related to their ability to detect and manage adverse situations, such as suicidal tendencies and self-harm behavior. This is related to their perception of the technological limitations in terms of understanding and interpreting the intent of the user. CAs also might not be able to provide personalized information related to the user’s specific or unique circumstances. However, some mental health supporters also shared pertinent considerations regarding what a CA should say as it may not be immediately able to identify the user reactions to their statements. One mental health supporter further suggested having discussions with patients with mental health issues themselves to understand the kind of statements that “they don’t like to hear or statements that really helped them feel like they’re being listened to” (Mental health supporter 017). To address these issues some highlighted the importance of having professional support in-app to handle potential mental health crises or providing users with the resources to seek professional mental health services.

### Cultural Relevance in the Design and Development of the mHealth Intervention

The following two themes related to the broader context of the mHealth intervention emerged: (1) the inclusion of shared local narratives and (2) the importance of global context. Participants mentioned the importance of including shared narratives within the Singapore context. For instance, a mental health supporter mentioned that local students’ concerns typically revolved around their academic performance, career-related concerns, and “performance indicators”–related pressures (Mental health supporter 005).

In the Asian community, it’s pretty interesting, when each time they go and see a counsellor, it’s always [about] 2 main issues, one is academic performance and then another career-related. It is always a performance indicator kind of stuff right.

Mental health supporter 005

Some suggested the inclusion of specific common experiences that could serve as a relatable experience for students on their mental health journey such as the National Service, the mandatory conscription for every male citizen and permanent resident in Singapore.

However, some mental health supporters also mentioned the need to position the app within the wider global community as they perceived Singapore to be a more globalized society with similar problems across various cultures in the world. Although there could be a need to include Singapore-specific content, they also expressed the need to include some considerations in terms of finding the common ground between the various cultural groups within and beyond the Singapore context.

Regarding language considerations, students and mental health supporters expressed the preference to include more colloquial words and slang in the mHealth intervention. This was seen as important to make the intervention more relatable to users (Mental health supporter 019). Finally, in terms of the words and phrases used in the apps, the mental health supporters also shared pertinent considerations regarding what an app should and should not say, as a chatbot could not immediately gauge the user’s reactions to their statements.

## Discussion

### Principal Findings

This study highlighted the various needs and considerations related to the preferred functions and acceptance of mHealth intervention from university students and mental health supporters in Singapore. We identified various themes related to the perception of a healthy lifestyle, mental health services and campaigns, and existing mHealth interventions. Furthermore, we also identified barriers to participating in mental health services available to students, such as prevalent stigma, accessibility of these services, and their personal preferences regarding help-seeking behavior. Students and mental health supporters also shared various recommendations to make the mHealth intervention more engaging through features such as gamification and story-based designs. As part of the discussion focused specifically on CA-delivered mHealth interventions, participants also shared some reservations about CA regarding its robotic and artificial nature. The mental health supporters also expressed concern about the CA’s ability to accurately interpret user input, which could cause safety and ethical concerns when mishandled compared with that of human support. Other recommendations include having in-app human support to allay these concerns. Finally, we also identified cultural considerations specific to university students in Singapore related to designing mHealth interventions for them.

Participants’ perceptions of healthy lifestyles and their experiences with existing digital health interventions provided valuable information on the possible barriers and facilitators involved in the development of mHealth intervention. The students in our study viewed health as a complex balancing act between various aspects of health, including physical, mental, and social health. An intervention focusing solely on 1 aspect of health, such as mental health, is less attractive compared with one that viewed mental health within the wider context of the lifestyles of students. This may include contextualizing stress within the context of balancing coursework and maintaining healthy relationships with their peers. Thus, our study suggests a preference for a holistic intervention as recommended by the World Health Organization [10]. However, a holistic digital intervention is rare, despite evidence that these aspects of health are highly interconnected [35]. It is important, however, to contextualize participants’ suggestions to recognize users’ immediate needs within the discussions. For example, students who expressed the importance of socialization as an integral element of the intervention also expressed the feeling of social isolation because of the pandemic restrictions during the discussion. This is also evident in many other studies conducted during the pandemic [36].

Most of the feature suggestions by participants were not culturally specific to either university students or the Singaporean context. Features such as personalization, reminders, and gamification, as mentioned above, have previously been recommended as necessary engagement features to increase adherence to interventions [20,21]. These may suggest that users would minimally expect these features to be implemented based on their previous experience with other apps in the market. Participants expressed a preference for the inclusion of culturally specific language, such as colloquial words and local slang in the mHealth intervention to improve relatability and engagement. This is an important consideration for future mHealth interventions in Singapore, as the acceptability and use of interventions tend to be guided by existing social and cultural norms [37]. These findings also highlight the importance of engaging end users in the discussion of language and other culturally specific preferences as part of participatory design [38,39] to better tailor interventions to suit user needs.

Within the context of a CA-delivered intervention, we found that personalized feedback and suggestions based on users’ needs and characteristics are critical to users. Both students and mental health supporters expressed the importance of CA being responsive and not responding in a robot-like manner. However, they both recognized that the intervention could provide complementary services such as providing immediate feedback or support at any time of the day [40]. A recent review also found high perceived usefulness of CA interventions but shared similar concerns related to the CA’s linguistic capabilities [40]. This suggests the importance of positioning mHealth interventions, not as a replacement for humans but as complementary services to health care services.

Our result also suggested that the fear of mental health stigma played an important role in preventing students and young adults from seeking mental health services. The link between mental health stigma and help-seeking behavior has been extensively documented globally [29,41]. Issues such as privacy concerns and the fear of judgment from others were also present in our study, similar to other qualitative studies within the local context [29,42]. However, many participants expressed positive attitudes toward mental health initiatives launched during the pandemic. For instance, students expressed that these campaigns normalize mental health conditions, especially among their peers. A recent study on the antistigma initiative in the local university context also suggests that a single 50-minute intervention session could reduce mental health stigma toward depression, measured using the Community Attitude to Mental Illness scale [43]. Such an intervention can also be delivered via mHealth as mHealth intervention may be as effective as information pamphlets with the added advantage of being less stigmatizing, easier to access, and more trustworthy than pamphlets [44].

Both major stakeholders acknowledged that help-seeking behavior among university students is lacking. This was supported by a recent mental health survey in Singapore, which found that more than one-third of those with mental health conditions never sought help for their mental health issues in their lives [5]. Our study, however, found a much more complex structure of support among our participants. Many of our participants expressed a stepwise approach to their mental health support starting from immediate friends and family members or self-exploration. Although many expressed reservations about seeking professional mental health care, they were not completely against seeking professional help when their immediate support was completely exhausted. This presents an opportunity for mHealth interventions to provide bridging services between users and professional mental health, as studies have found the potential of CA to provide peer support similar to human support [45,46].

Our study recommends the implementation of mHealth with CA-delivered intervention as a buffer to act as a peer supporter and a pathway to connect with professional services. An example would be to implement such interventions within the learning communities of higher learning institutions, where students are placed in smaller groups to foster communication between students and faculty [47]. Within such systems, mHealth interventions can be easily assigned to and supported by the groups and can be used for early detection and to facilitate access to mental health care. Although university students in our samples generally preferred their friends and family, the intervention can provide an intermediary shield to increase mental health resilience for those in dire need of mental health services and healthy individuals. mHealth intervention with human support may be beneficial as it can be flexibly deployed as a stand-alone intervention or used to notify health care professionals when the current form of support is not sufficient [48]. At present, most mHealth interventions are typically delivered as stand-alone interventions, unsupported by humans, and not embedded within the health care system [16]. Although the scalability of an unguided and fully automated intervention is very promising [49], including human elements within the intervention may allay many of the concerns expressed by users about the technological limitations of the system [50]. In addition, therapists perceived blended treatment to have fewer disadvantages than stand-alone digital interventions because of the lack of therapeutic process components in stand-alone interventions such as the lack of nonverbal signals and missing contextual information [51]. Some studies have also shown that including human support in mHealth may be effective in reducing mental health symptoms and improving user engagement in the intervention [52,53], although these findings are still preliminary [54].

### Recommendation for the Improvement of the mHealth Intervention

On the basis of the participation of participants, the following 3 major recommendations were outlined to improve the proposed mHealth intervention: *the delivery format of the intervention*, *the participation characteristics of the intervention,* and *the presentation of the CA within the intervention.* Table 4 describes the perceived barriers and facilitating factors based on their experience with other mHealth apps, and opportunities for specific feature recommendations derived from the FGDs and interviews.

**Table 4:** Opportunities for conversational agent (CA) or mobile health interventions by areas of improvement based on barriers and facilitating factors mentioned by participants.

Areas of improvement	Barriers	Facilitators	Feature recommendations	Content recommendations
The delivery format of the intervention (eg, stand-alone only or with human support [blended])	Lack of “human touch” in CA-delivered intervention	The perception that the intervention could be used as a bridging service toward professional mental health support	Remote human support in-app (hybrid system) and integrating the intervention within the health care system	Include referrals information to public or private mental-health institutions
Engagement feature of the intervention (eg, gamification, rewards, reminders, and story-based design)	Lack of motivation to engage in healthy lifestyle behavior	Participants’ preference for novel and intrinsic elements in CA-delivered intervention	Gamification elements, eg, a point-based system for completing modules	Mindfulness, CBT^a^, psychoeducation content, transdiagnostic approach, and blended approach
Engagement feature of the intervention (eg, gamification, rewards, reminders, and story-based design)	Stigma in mental health	The normalization of mental health issues through public mental health campaigns	Including shared narratives through story-driven intervention	Localized and contextualized narratives such as transitioning to university or working adult life
Presentation of the CA within the intervention (eg, tone and content of the dialogues)	The CA appeared cold and unemotional	The normalization of mental health issues through public mental health campaigns	Having an avatar or visual representation of the CA	Inclusion of colloquialism and local slang (“Singlish”) and defining the CA’s personality and conversation style

^a^CBT: cognitive behavioral therapy.

### Strengths and Limitations

This study involved various stakeholders from potential users to mental health supporters from various professions, including faculty members, counselors, psychologists, a clinical psychologist, and a psychiatrist. Although the study used purposeful sampling to gather views and opinions from various mental health professionals, these perspectives were valuable to understanding the various unique systems and how the students engaged these systems for mental health support. The convergence of opinions among mental health professionals suggested agreement between the various professions on the needs and barriers to help-seeking behavior among university students. Opinions on the mHealth intervention between students and mental health supporters also largely converged with more detailed therapeutic concepts used by supporters compared with those of students. Having external facilitators also helped reduce the confirmation bias by not emphasizing specific features that the research team was currently exploring and instead allowing open discussion between the facilitators and FGD participants. However, because both facilitators and FGD participants were not experts in the field of mHealth intervention design, there was also a lack of depth in the discussion of the pros and cons of specific features mentioned during the discussion. However, current findings are limited to the context of mental health support among university students and generalizability is limited owing to the qualitative nature of the study.

### Conclusions

This work presents valuable insights and contextual evidence on the preferences of university students in Singapore regarding the mHealth intervention for the prevention of CMDs through formal qualitative analysis. We found potential barriers and factors that can facilitate the users’ engagement with the intervention. We also provided specific design recommendations within the context of Singaporean university students based on our discussions to address the barriers identified. Students felt that mental-health stigma was still prevalent, although the pandemic had slowly shifted the sentiment, especially among their peers. However, they generally were reluctant to approach professional mental health services and preferred their friends and family, which showed the potential of a scalable mHealth intervention, which has been previously shown to bridge the gap between users and mental health care providers. Although students and mental health supporters were reluctant to use mHealth technologies such as CA in the prevention of CMDs, they saw these interventions as part of a blended intervention with hybrid support from both technology and humans. We also provided recommendations for improving engagement in future mHealth interventions.

## Appendix

Multimedia Appendix 1 Focus group discussions participant survey.

Multimedia Appendix 2 Focus group discussion interview guide.

Multimedia Appendix 3 Mental health supporter participant survey.

Multimedia Appendix 4 Mental health supporter interview guide.

Multimedia Appendix 5 The Consolidated Criteria for Reporting Qualitative Research checklist.

Multimedia Appendix 6 Themes, subthemes, and participants' most representative quotations.

## Abbreviations

CA: conversational agent
CMD: common mental disorder
FGD: focus group discussion
mHealth: mobile health
