# Supplementary material for: Exploring the Perceptions of mHealth Interventions for the Prevention of Common Mental Disorders in University Students in Singapore: Qualitative Study
Source: J Med Internet Res. 2023 Mar 20;25:e44542. doi: 10.2196/44542 (PMC10131767; doi:10.2196/44542)
Supplement: Multimedia Appendix 1 [file jmir_v25i1e44542_app1.docx]

| In order to facilitate the focus group discussions, please complete the following questionnaires. | | | | |
| --- | --- | --- | --- | --- |
|  | | | | |
| **Section A: Sociodemographic and Clinical Questionnaire** | | | | |
| 1. Please state your gender: | | | | |
| ☐ Male | | | | |
| ☐ Female | | | | |
|  | | | | |
| 1. Please state your ethnicity: | | | | |
| ☐Chinese | | | | |
| ☐Malay | | | | |
| ☐Indian | | | | |
| ☐Others | | | | |
| Please state: ___________________________ | | | | |
|  | | | | |
| 1. Current Course of Study: | | | | |
| ☐ Undergraduate (Degree) | | | | |
| ☐ Postgraduate degree (Masters/PhD) | | | | |
|  | | | | |
| Please state your major: ______________________ | | | | |
|  | | | | |
| 1. How confident are you with using smartphone devices (e.g. searching for information on the phone, downloading applications, using messaging apps such as Telegram, Whatsapp, etc) | | | | |
| ☐ Very confident | | | | |
| ☐ Confident | | | | |
| ☐ Average | | | | |
| ☐ Mildly confident | | | | |
| ☐ Not confident | | | | |
|  | | | | |
| 5.How often over the last two (2) weeks have you felt down, depressed, or gotten little enjoyment from doing things?  (Use “X” to indicate your answer) | Not at all  0 | Several  Days  1 | More  than half  the days  2 | Nearly  every  day  3 |
| 6. How often over the last two (2) weeks where you bothered by feeling anxious, nervous, on edge, or unable to stop or control worrying? (Use “X” to indicate your answer) | Not at all  0 | Several  Days  1 | More  than half  the days  2 | Nearly  every  day  3 |
|  |  |  |  |  |
|  |  |  |  |  |
|  |  |  |  |  |
| **Section B: Preliminary Questions** | | | | |
| 1. For the four words or phrases below (a-d), please type in the **first three** words that come to mind in the corresponding spaces. Remember that there are no right or wrong answers and to be as honest as possible with your responses. 2. Healthy Lifestyle behaviour   1)__________________________________________________________  2)__________________________________________________________  3)__________________________________________________________ | | | | |
| 1. Mental Health   1)__________________________________________________________  2)__________________________________________________________  3)__________________________________________________________ | | | | |
| 1. Depression   1)__________________________________________________________  2)__________________________________________________________  3)__________________________________________________________ | | | | |
| 1. Anxiety 2. _________________________________________________________ 3. _________________________________________________________ 4. _________________________________________________________ | | | | |
| 1. Digital Health Interventions   1)__________________________________________________________  2)__________________________________________________________  3) _________________________________________________________ | | | | |
| 1. Chatbot/conversational agent   1)__________________________________________________________  2)__________________________________________________________  3)__________________________________________________________ | | | | |
|  | | | | |
| 1. Previous research has shown that people in Singapore react in similar ways about members of the community who have a mental illness. Below are some of the statements that reflect these findings.   Please read each of these statements and indicate whether you agree or disagree | | | | |
|  | | | | |
| **Statement** | **Agree/Disagree** | | | |
| People with mental illness are dangerous | Agree/Disagree | | | |
| People with mental illness are incompetent at work | Agree/Disagree | | | |
| People with mental illness are a burden to society | Agree/Disagree | | | |
| Mental illnesses are medical conditions | Agree/Disagree | | | |
| I feel comfortable communicating with someone who has a mental illness | Agree/Disagree | | | |
| You have now come to the end of the survey!  Thank you very much for taking the time to answer these questions. A member of our research team will get back to you on your eligibility to participate in the focus group discussions and next steps. We may also contact you to clarify some of the responses to this survey if necessary, to ensure that the right decisions with regard to participation suitability are being made    In the meantime, if you have any questions about this survey or the focus group, you may contact the following members of the research team:  Principal investigator: Asst Prof Lorainne Tudor Car \|Lee Kong Chian School of Medicine, Nanyang Technological University (NTU) \| ([lorainne.tudor.car@ntu.edu.sg](mailto:lorainne.tudor.car@ntu.edu.sg) )  Postdoctoral researcher: Dr Alicia Salamanca \| Future Health Technologies, Singapore-ETH Centre \| ([alicia.salamanca@sec.ethz.ch](mailto:alicia.salamanca@sec.ethz.ch)) | | | | |
