# Supplementary material for: Exploring the Perceptions of mHealth Interventions for the Prevention of Common Mental Disorders in University Students in Singapore: Qualitative Study
Source: J Med Internet Res. 2023 Mar 20;25:e44542. doi: 10.2196/44542 (PMC10131767; doi:10.2196/44542)
Supplement: Multimedia Appendix 2 [file jmir_v25i1e44542_app2.docx]

**Introduction**

Research team and facilitators roles and background

Study description

FGDs details

**Warm up**

Name

What do you do?

What do you must look forward to in your normal daily activities?

**Healthy Living**

What is your normal daily routine like? (Chat box)

What is a healthy lifestyle to you?

What stops you from making healthier lifestyle choices?

If you had a choice, what could you do differently to improve what you are doing now? Chat box)

**Perceptions of lifestyle and Mental Health**

What expressions will you use to describe a person with depression/anxiety?

What do you think are the causes of anxiety and/or depression?

Name some mental health awareness activities/programs in Singapore (chat box)

What do you think of the mental health awareness activities/programs in Singapore?

How useful do you think they are?

If you were seeking support for healthy lifestyle including mental health where/who would you go to? (Chat box)

**Technology & Digital health**

What is your experience in using lifestyle apps/mental health apps and technologies?

What features would you like to see in this?

**Digital Lifestyle Coaching/Chatbot Apps**

Description of smartphone-based chatbot intervention including lifestyle and mental health:

- Imagine…an App that:
  - Provides personalized digital coaching on a variety of health topics, delivered at your fingertips from the safety of your very home.
  - Keeps you up to date with guidelines and what practical steps we can take specifically for your health (including physical activity and diet & well-being.
  - Provides mental health support to cope with depression, anxiety, and sleep difficulties.
  - It is created by an expert team of therapists and mental health researchers.
- Some lifestyle smartphone apps contain digital health coaches. These digital health coaches are designed by health professionals to simulate human conversations with people to provide health support. In other words, they are designed to communicate with you like an assistant to a doctor or a psychologist.

What are your first impressions of such apps with digital health coaches?

How can we encourage people to use such an app long term? (Chat box).

What are your views on sharing health data *(like heart rate, or step counts)* tracked by an activity tracker or a smartphone?

**Cooling down questions**

Summation (checking)
