# Supplementary material for: Exploring the Perceptions of mHealth Interventions for the Prevention of Common Mental Disorders in University Students in Singapore: Qualitative Study
Source: J Med Internet Res. 2023 Mar 20;25:e44542. doi: 10.2196/44542 (PMC10131767; doi:10.2196/44542)
Supplement: Multimedia Appendix 3 [file jmir_v25i1e44542_app3.docx]

**Multimedia appendix 3: Mental health supporter participant survey**

| **Survey questions for experts and stakeholders on smartphone-based chatbot-delivered intervention to prevent depression/anxiety in university students in Singapore** |
| --- |
| **Section A:** Section A collects demographic information and general information regarding use and attitudes towards technology |
| *For questions 1-5 please tick in the boxes where indicated. Please ensure that only one box is ticked per question.* |
| 1. Gender:   ☐ Male  ☐ Female |
|  |
| 1. Age   ☐ < 25 - 45  ☐ 45 + |
|  |
| 1. Years of experience:   ☐ <1 – 2 years  ☐ 3 – 5 years  ☐ 6 – 10 years  ☐ >10 years |
|  |
| 1. Profession:   ☐ Clinical Psychologist  ☐ Counsellor  ☐ Social Worker  ☐ Psychiatrist  ☐ Clinical Psychologist  ☐ Digital Health Worker  ☐ Academic  ☐ Others, please state: _____________ |
| 1. What is your highest academic degree?   ☐ Certificate or Diploma  ☐ Bachelor’s degree  ☐ PhD  ☐ Master’s degree  ☐ Doctoral Degree  ☐ Others  Please state: _____________________ |

|  | | | | | | | | | | | |
| --- | --- | --- | --- | --- | --- | --- | --- | --- | --- | --- | --- |
| *For questions 6 and 7 please indicate a number from 1 to 7 that corresponds to answers to the respective questions.* | | | | | | | | | | | |
|  | | | | | | | | | | | |
|  | | | | | | | | | | | |
| 1. How would you rate your technological competence | | | | | | | | | | | |
| Novice |  |  |  | |  | | |  | | Expert | |
| 1 | 2 | 3 | 4 | | 5 | | | 6 | | 7 | |
|  | | | | | | | | | | | |
| 1. How helpful do you find technology to be in your everyday life? | | | | | | | | | | | |
| Not  helpful |  |  |  | |  | |  | | Very helpful | | |
| 1 | 2 | 3 | 4 | | 5 | | 6 | | 7 | | |
|  | | | | | | | | | | | |
| Section B: Section B collects specific information exploring the mental health landscape for university students in Singapore, and how digital interventions can be used and designed to meet the needs of end-users and other stakeholders | | | | | | | | | | | |
|  | | | | | | | | | | | |
| For questions 8-11 please type the responses to the questions on the lines provided and tick in the boxes where indicated | | | | | | | | | | | |
|  | | | | | | | | | | | |
| 1. What are the common mental health issues that university students in Singapore present with? (tick all that apply)   ☐ Depression  ☐ Anxiety  ☐ Substance abuse  ☐ Eating disorder  ☐ Psychosis  ☐ Self-harm | | | | | | | | | | | |
|  | | | | | | | | | | | |
| 1. If there are any others that are not mentioned, please list them below:   ____________________________________________________________________________________________________________________________________________________________________________________________________________________________________________________________________________ | | | | | | | | | | | |
|  | | | | | | | | | | | |
| 1. What is a typical presentation of a university student in Singapore with depression/anxiety?   ____________________________________________________________________________________________________________________________________________________________________________________________________________________________________________________________________________ | | | | | | | | | | | |
| 1. What are the barriers that university students in Singapore face when it comes to seeking support for mental health issues?   ____________________________________________________________________________________________________________________________________________________________________________________________________________________________________________________________________________ | | | | | | | | | | | |
| For questions 12-17 please indicate a number from -3 to +3 that corresponds to your attitudes and feelings regarding the respective statements | | | | | | | | | | | |
|  | | | | | | | | | | | |
| 1. I find the use of digital interventions for mental health to be: | | | | | | | | | | | |
| Harmful |  |  | |  | |  | |  | | | Beneficial |
| -3 | -2 | -1 | | 0 | | 1 | | 2 | | | 3 |
|  | | | | | | | | | | | |
| 1. I find digital interventions for mental health to be: | | | | | | | | | | | |
| Not Credible |  |  | |  | |  | |  | | | Credible |
| -3 | -2 | -1 | | 0 | | 1 | | 2 | | | 3 |
|  | | | | | | | | | | | |
| 1. In my work I use digital tools to search for disorder or treatment-related information | | | | | | | | | | | |
| Totally Disagree |  |  | |  | |  | |  | | | Totally Agree |
| -3 | -2 | -1 | | 0 | | 1 | | 2 | | | 3 |
|  | | | | | | | | | | | |
| 1. In my work I refer websites or web-based programs related to mental health/health to my clients | | | | | | | | | | | |
| Totally Disagree |  |  | |  | |  | |  | | | Totally Agree |
| -3 | -2 | -1 | | 0 | | 1 | | 2 | | | 3 |
|  | | | | | | | | | | | |
| 1. In my work I refer mobile health applications to my clients | | | | | | | | | | | |
| Totally Disagree |  |  |  | |  | | |  | | Totally Agree | |
| -3 | -2 | -1 | 0 | | 1 | | | 2 | | 3 | |
|  | | | | | | | | | | | |
| 1. In my work I use digital tools to communicate with patients | | | | | | | | | | | |
| Totally Disagree |  |  |  | |  | | |  | | Totally Agree | |
| -3 | -2 | -1 | 0 | | 1 | | | 2 | | 3 | |
|  | | | | | | | | | | | |
| *You have now come to the end of this survey. Thank you for taking the time to answer these questions. Your responses are very valuable for our research in developing a digital health coach for preventing depression and anxiety.* | | | | | | | | | | | |
