# Supplementary material for: Exploring the Perceptions of mHealth Interventions for the Prevention of Common Mental Disorders in University Students in Singapore: Qualitative Study
Source: J Med Internet Res. 2023 Mar 20;25:e44542. doi: 10.2196/44542 (PMC10131767; doi:10.2196/44542)
Supplement: Multimedia Appendix 4 [file jmir_v25i1e44542_app4.docx]

**Introduction**

Interviewer background

Study description

Interview details

**Warm up**

Participant current role

Participant experience

Participant relevant experience in the field of prevention CMDs in Singapore

**CMDs and Digital health interventions**

Typical patient/ client with CMDs

Digital interventions in the field of CMDs

Digital interventions supporting university students

Concerns regarding use of SBIs in mental health (CMDs)

Experience and knowledge about digital interventions in mental health

(Recommended apps to clients, features preferences)

**Smartphone-based chatbot intervention**

Perception of the conceptual model (research team)

Therapeutic components and features

**Integrating Smartphone-based interventions into the Singapore mental healthcare system**

Typical pathways to mental health support for a university student

Efforts on a system level to integrate digital solutions into the therapeutic work processes

Implementation and integration of Smartphone-based interventions into the healthcare system (facilitators and barriers)

Digital interventions in the field of preventions of CMDs

**Cooling down questions**

Summation (checking)
