# Supplementary material for: Exploring the Perceptions of mHealth Interventions for the Prevention of Common Mental Disorders in University Students in Singapore: Qualitative Study
Source: J Med Internet Res. 2023 Mar 20;25:e44542. doi: 10.2196/44542 (PMC10131767; doi:10.2196/44542)
Supplement: Multimedia Appendix 5 [file jmir_v25i1e44542_app5.pdf]

Supplementary material 1: The consolidated criteria for reporting qualitative research (CORE-Q)

COREQ 32-item checklist

**No**	**Item**	**Guide questions/ description**	**Response/Reported on page (#)**
**Domain 1: Research team and reflexivity**			
Personal Characteristics			
1.	Interviewer/facilitator	Which author/s conducted the interview or focus group?	Focus groups were conducted by external facilitators. Interviews were conducted by three authors: AS, AA, AIJ (Page 7).
2.	Credentials	What were the researcher's credentials? *E.g. PhD, MD*	AS had a PhD, AA and AIJ had a MSc, and XL had a BA (Page 1).
3.	Occupation	What was their occupation at the time of the study?	AS was a postdoctoral researcher, AA was a PhD researcher, and AIJ and XL were research assistants (Page 1).
4.	Gender	Was the researcher male or female?	AS, AA, and XL were female. AIJ was male.
5.	Experience and training	What experience or training did the researcher have?	AS and AIJ had experience in carrying out qualitative research. (Page 9)<br>AA and AIJ had been trained to conduct interviews. (Page 9)
Relationship with participants			
6.	Relationship established	Was a relationship established prior to study commencement?	Some experts were known contacts of AS, AA, and LTC (Page 6).
7.	Participant knowledge of the interviewer	What did the participants know about the researcher? e.*g. personal goals, reasons for doing the research*	Name, occupation, reasons for doing the research were detailed in the informed consent (Page 6)
8.	Interviewer characteristics	What characteristics were reported about the interviewer/facilitator? e.g. *Bias, assumptions, reasons and interests in the research topic*	Name, occupation, reasons for doing the research for the researchers were detailed in the informed consent (Page 6). The facilitators verbally explained their relationship to the project during the introduction phase of

No	Item	Guide questions/ description	Response/Reported on page (#)
			the FGD (Page 7)
**Domain 2: study design**			
Theoretical framework			
9.	Methodological orientation and Theory	What methodological orientation was stated to underpin the study? *e.g. grounded theory, discourse analysis, ethnography, phenomenology, content analysis*	Thematic analysis approach (Page 7-8).
Participant selection			
10.	Sampling	How were participants selected? *e.g. purposive, convenience, consecutive, snowball*	Convenience sampling, snowball sampling, and purposive sampling (Page 6).
11.	Method of approach	How were participants approached? e*.g. face-to-face, telephone, mail, email*	Email and via social media (Page 6).
12.	Sample size	How many participants were in the study?	30 university students in the focus groups, 11 mental health supporters for the interviews (Page 11-12).
13.	Non-participation	How many people refused to participate or dropped out? Reasons?	NA. Participants opt-in for the FGDs/interviews
Setting			
14.	Setting of data collection	Where was the data collected? e*.g. home, clinic, workplace*	Focus groups and interviews took place online via Zoom, a videoconferencing platform (Page 6).
15.	Presence of non-participants	Was anyone else present besides the participants and researchers?	External moderators were there for the FGDs (Page 7).
16.	Description of sample	What are the important characteristics of the sample? *e.g. demographic data, date*	University undergraduate students and experts with at least one years' experience working with young adults with depression or anxiety (Page 10-11).

No	Item	Guide questions/ description	Response/Reported on page (#)
Data collection			
17.	Interview guide	Were questions, prompts, guides provided by the authors? Was it pilot tested?	Yes. Yes. (Appendix 4 and 5).
18.	Repeat interviews	Were repeat interviews carried out? If yes, how many?	No.
19.	Audio/visual recording	Did the research use audio or visual recording to collect the data?	Audio recording (Page 7).
20.	Field notes	Were field notes made during and/or after the interview or focus group?	Yes. During. (Page 7).
21.	Duration	What was the duration of the interviews or focus group?	Focus groups were 90 minutes and interviews were 45-60 minutes (Page 7).
22.	Data saturation	Was data saturation discussed?	Yes. (Page 7)
23.	Transcripts returned	Were transcripts returned to participants for comment and/or correction?	No.
**Domain 3: analysis and findings**			
Data analysis			
24.	Number of data coders	How many data coders coded the data?	Two (AI and XL) (Page 7-9).
25.	Description of the coding tree	Did authors provide a description of the coding tree?	Yes (Pages 7-10).
26.	Derivation of themes	Were themes identified in advance or derived from the data?	Themes were derived from both methods (Page 7-10).
27.	Software	What software, if applicable, was used to manage the data?	ATLAS.ti V9 (Page 7).
28.	Participant checking	Did participants provide feedback on the findings?	No (page 10)

No	Item	Guide questions/ description	Response/Reported on page (#)
Reporting			
29.	Quotations presented	Were participant quotations presented to illustrate the themes / findings? Was each quotation identified? e.*g. participant number*	Yes. Yes. (Multimedia Appendix 6)
30.	Data and findings consistent	Was there consistency between the data presented and the findings?	Yes (Pages 12-20).
31.	Clarity of major themes	Were major themes clearly presented in the findings?	Yes (Pages 12-20).
32.	Clarity of minor themes	Is there a description of diverse cases or discussion of minor themes?	Yes (Pages 12-20).

The COREQ aims to support the transparency of all aspects of qualitative research by providing clear standards for reporting qualitative research. The rationale should briefly discuss the justification for choosing that theory, approach, method, or technique rather than other options available, the assumptions and limitations implicit in those choices, and how those choices influence study conclusions and transferability.

Reference:

1. Booth, Andrew, Karin Hannes, Angela Harden, Jane Noyes, and Janet Harris. "COREQ (consolidated criteria for reporting qualitative studies)." (2014): 1-320.
