# Supplementary material for: Exploring the Perceptions of mHealth Interventions for the Prevention of Common Mental Disorders in University Students in Singapore: Qualitative Study
Source: J Med Internet Res. 2023 Mar 20;25:e44542. doi: 10.2196/44542 (PMC10131767; doi:10.2196/44542)
Supplement: Multimedia Appendix 6 [file jmir_v25i1e44542_app6.docx]

|  |
| --- |
| Theme 1: Perception and barriers to healthy lifestyle as students |
| **Healthy lifestyle** |
| “I think that a healthy lifestyle will be across all the aspects of health that could possibly be in life. So like: physical, social, emotional, cognitive health” |
| *FGD1, Student 001* |
| **Lack of motivation** |
| “Even if I have [the] time to do my exercise but sometimes, I'm just a bit like lazy.” |
| *FGD2, Student 035* |
| **Balanced lifestyle** |
| “Yeah, I think to me what's a healthy lifestyle it's two things. Mainly …work-life balance. So, a good balance between school [and] work. And also I think personal interest and time for yourself” |
| *FGD4, Student 015* |
| **Having other commitments** |
| “Then what stops me from making healthier lifestyle choices would be, sometimes I have obligations to fulfil. For example, I have work to do, but the same time I wanted I go out with my friends … so I end up like losing sleep or my mealtimes become irregular” |
| *FGD1, Student 001* |
| **Mental health** |
| “It's also important to take care of our mental wellbeing. So, for example sometimes I do feel a bit isolated due to the pandemic. I find myself alone at home most of the time, instead of hanging out with my friends as usual, yeah. So, to me, having a healthy lifestyle also includes being aware of how you are feeling and addressing whatever concerns you may have” |
| *FGD6, Student 008* |
| **Unhealthy habits** |
| “I think because for me it's like [my] bad habit tends to be more towards the mental side because like... yeah. For example, if I- if something goes wrong, it's like I usually jump towards blaming myself. So that's like something that uh I'm trying to work like on it yeah.” |
| *FGD4, Student 025* |
| **Social health** |
| “I think healthy lifestyle to me [is] more than physical activities. To me, it has [the] meaning of having appropriate- decent number of social time… What stops me from making healthier life choices, I think it’s definitely the group size restriction and whole pandemic. Very disruptive, cannot go out with more than five, … so I imagine, most of us eventually just decided to stay indoors and meet up less frequently.” |
| *FGD1, Student 033* |
| **COVID -19 Pandemic** |
| “I think definitely the group size restriction and whole pandemic, very disruptive, cannot go out with more than five, but then again, if we do, we kind of split ourselves apart, we still kind of go out together still and somehow, so there is the – the fear that we might get caught [laughs], so I imagine, most of us eventually just decided to stay indoors and meet up less frequently.” |
| *FGD1, Student 033* |
| “Definitely there are increased levels of anxiety, stress, and depression, some of them I think they have had home-based learning or, you know, studying online. Some people find it quite hard to focus when they are left on their own, they rather go to classes, go to lectures, things like that. Isolation is a big part with Covid, a lot of them cannot really hang out in groups with their friends or do sports together. So, they end up like you know, having to reach out to one, one other friend to go out, but then that friend might be already booked by another friend, so they kind of feel quite lonely, yeah” |
| *Mental health stakeholder 011, Psychologist* |
| Theme 2: Accessibility and trust in mental health services |
| Subtheme 2.1: Accessibility of mental health services |
| **Preference for friends and family** |
| “I put friends because they [are at the] same point [of time] in my life. So, my experience will be kind of like similar to theirs. So maybe they kind of understand me better. Then I just maybe seek their opinions. But if the problem persists, then I might go to my family then” |
| *FGD1, Student 016* |
| **Preference for self-exploration** |
| "I think [I] would also look at online resources such as YouTube videos or online forums to see how … how others react to it? And perhaps go to specific online communities that is geared towards mental health to talk to people." |
| *FGD6, Student 018* |
| **Access to mental health services** |
| “For me, going to therapy … is kind of a step further away because, I’m not sure if it's expensive. I think it's expensive. And probably too much of a hassle if we have sufficient resources online yeah.” |
| *FGD6, Student 008* |
| Subtheme 2.2: Stigma surrounding CMD |
| **Stigma in mental health** |
| “For me, I'll just most likely talk to friends who I’ve already established like a connection with, and like we’ve already talked about mental health issues before, because I feel that I’ll receive less stigma than trying to talk to someone new” |
| *FGD3, Student 009* |
| “I imagine almost every country in the world has its problems in this way [related to mental health stigma], but I think what the struggle with within Singapore is that it is, it is worse than in other places, and it is harder for people to be open.” |
| *Mental health stakeholder 006, Faculty member* |
| Subtheme 2.3: Relatability and trust |
| **Therapy only for serious issues** |
| “If it [mental health issues/symptoms] really gets very serious, then that’s when I’ll take like therapy or counselling, where it’s like a, you know, third party.” |
| *FGD6, Student 013* |
| Most of the time I will approach my friends first, ‘cause like I hang out with them more, they probably know me better in that sense, or they can relate to the problems I faced. Then, maybe next will go to my – my siblings… when both friends and family can’t help, then I might go to counsellor |
| *FGD3, Student 032* |
| **Lack of receptiveness and trust in mental health services** |
| “I have a friend who tried to like seek a counsellor, like try to see a counsellor in [school], but like the counsellor was like very insensitive. So like, it kind of like makes me don’t really want to seek, seek like a coun – for a counsellor in school” |
| *FGD5, Student 034* |
| “So I think that that stigma is something that stops people asking for help when uhm when they need it because they're worried that that mean it's going to go on their record a big sense of if I seek mental health treatment, I need to declare it to my workplace and people are going to know it's going to affect my insurance, is going to [affect] my employability it's going to affect how people see me” |
| *Mental health stakeholder 004, Faculty member* |
| Theme 3: Reach and relevance of mental health campaigns |
| **Accessibility of mental health campaigns** |
| “[Campaigns] does provide like an outlet for like students, at least like the student population who may need to, to know that there is another outlet for them to like share, for example, they want to seek counselling or if they feel like they might want to get diagnosed or something like that um.” |
| *FGD5, Student 041* |
| **Promote awareness of mental health and normalise issues** |
| “because of the pandemic situation, more of, more of like, I guess, more in terms of the- [online] community has spread – helped spread or market this program, so I guess that helps as well” |
| *FGD6, Student 018* |
| “I think it's the same with so – social media right? When people use it [mental health resources] more, like talk about it, and then they use a lot of campaigns on social media. So when it gets to the news also, then there’s more awareness lah, yeah, so I think it’s always the awareness part [of mental health].” |
| *Mental health stakeholder 018, Clinical Psychologist* |
| **Normalizing mental health issues** |
| “I think the [mental health awareness] programs they help in terms of … they disproving the stigma of those with mental health issues.” |
| *FGD6, Student 018* |
| **Lack of appeal in mental health campaigns** |
| “[University] have this is like, they always send this email that like, saying there's this, uh, talks on mental wellbeing and so on, but I actually… I see already then I just delete. Because like I don't think it's relevant to me. So I think this actually doesn't really help to raise awareness or anything ah, like there – they are quite low profile in promoting the activities ah, just in my opinion.” |
| *FGD3, Student 032* |
| **Campaigns are ineffective in promoting help seeking behaviours** |
| “I honestly feel that the program in Singapore [are more secondary]. It relies too much on people around the person to realize and to sound out. Because I feel like whoever is struggling through this mental health issues right, they won't, they won't reach out first, you know? So it's the person around them who has to reach out.” |
| *FGD6, Student 012* |
| Theme 4: Preferred app engagement features |
| Subtheme 4.1: Basic app features |
| **App personalization** |
| “Yeah, but also depends on the individual as well, some people they want to find out more right [about the content], and want to have their – as in their level of willingness to change is higher and they want to do – they want to find out more, so those will be similar.” |
| *Mental health stakeholder 017, Mental Health First Aider* |
| **No paywall** |
| “The thing that's always put me off, is when they start when you just aren't getting [charged] you charge money for it [laugh] Because there's so yeah there's so many out there and the ones, you know they have either kind of free versions or you've got three bits at the beginning, and then you have to pay” |
| *Mental health stakeholder 004, Faculty member* |
| **Appealing UI** |
| “But I also hope that this would also be like an app, like a bit like aesthetic, I guess. Because like I felt like Lumi- for example *lumihealth* and uh *Wysa*, it’s like, they are good in the sense that they actually have very attractive UIs and so like, it's very approachable and it's very pleasing to the eyes. So like I think this what really like got people like interested in it.” |
| *FGD2, Student 021* |
| **Research-backed intervention** |
| “for some clients who seem to have certain mental health disorder, symptoms, or relapse, then letting them know that, okay, certain interventions that are tailored specifically for people with mental health disorders right, we may be able to get their buy-in more readily because they know that they are informed interventions that are specifically targeted for a special group of people, for those who actually have mental health issues” |
| *Mental health stakeholder 017, Mental Health First Aider* |
| **Ease of use** |
| “I guess something that I liked about these apps is that they are very simple. It's just every single day you go in and you log in your, your dailies. Whatever you are trying to do. So it's very simple. You go in, log in, then you log out.” |
| *FGD2, Student 005* |
| Subtheme 4.2: Active engagement features |
| **Gamification elements** |
| “Have an achievement reward system” |
| *FGD6, Student 012* |
| **Reminders/Prompts** |
| “I think like for long term usage maybe daily reminders at the start uhm so the inertia so you don't need to like remember it by yourself, so the app just reminds you to maybe use it” |
| *FGD4, Student 015* |
| **Story-based design** |
| “Perhaps like we can have like certain characters with like backstories to them, and so like, and then they, they have – and then they like, they know each other and so like, can sort of create this like this miniworld between the app so that players – people who use the app, they feel like their part of this – part of this world that is created in the app yeah” |
| *FGD2, Student 021* |
| **Guided activities** |
| “Interactive version where you can press like how many minutes you want to do or like which feeling you're feeling and then they will give you a customized meditation or a track based on that.” |
| *FGD6, Student 027* |
| **Mindfulness content** |
| I have tried a few like meditation mindfulness apps … And I've found that they're all really useful in a sense… But to a certain extent- I stopped using them because a lot of them have like, like, you need you need to unlock it with money |
| *FGD6, Student 027* |
| **CBT content** |
| “Maybe some of this homeworks can be built in together in there. For an example, the thought recording forms, and you know, maybe like breathing exercises, maybe couple of videos pertaining to that or you know, there’s this another technique called the hot cross buns, where it talks about a lot of things about the emotions, the bodily sensation, and things, so maybe these are these could already be there, could be built in together.” |
| *Mental health stakeholder 005, Counsellor* |
| **Social functions** |
| “I realized, and I think one of the things that could help keep that up is to have like social involvement like, um let's say I carry out an activity, I contribute to a group goal or something like that, like with my friends. Yeah I think that might help, I haven't really seen that in other apps before, yeah.” |
| *FGD6, Student 027* |
| **Expert support in app** |
| “I mean for mental health apps right, I wish to see a feature where you can actually speak to the healthcare professionals. So to people, people who are at the back and the other side. Not just bots.” |
| *FGD5, Student 044* |
| Subtheme 4.3: Self-awareness features |
| **Behaviour/feelings tracking functions** |
| “Also good to have trend checker - to be able to see entries on a rating scale over a period of time” |
| *FGD6, Student 031* |
| “So it's basically like a journaling thing, so you just type in what – you just check in and type what you feel on that day. So it’s usually like your own time own target.” |
| *FGD56, Student 018* |
| **Educational content** |
| “Just lifestyle mental health apps to give some types of like, like article, not say article but some educative, something to educate the user on. So whatever informative articles about, for example, eating healthy or like uh, the kind of stuff, this kind of apps will be useful to me.” |
| *FGD2, Student 020* |
| Theme 5: Barriers and facilitators of the proposed CA intervention |
|  |
| **Functions of the proposed CA intervention** |
| “[On using the intervention in a blended approach] Yeah I think that could be helpful, I think, as you know, assigning this sort of homework… [For example] we've discussed relaxation techniques this week, I want you to go home and over the next week, go through the relaxation exercises in the app as well, to try and reinforce what we've talked about today, or to track mood, because you know, there are clinical outcome measures that can take time to implement.” |
| *Mental health stakeholder 019, Associate Psychologist* |
| “[This] could be actively promoted to patient when they are waiting for their consultation at restructured hospitals so that they know that such a service exists and it's for- and they could actually explore it before they see their specialist” |
| *Mental health stakeholder 022, Psychiatrist* |
| “… So probably the way to work around this could be, if the app picks it up [suicidal ideation], I mean if the developer could really put in an algorithm and it picks it up, immediately encourage the client or anybody else to be referred to, to do a face-to-face counselling session.” |
| *Mental health stakeholder 005, Counsellor* |
| **“Humanness” of chatbot** |
| “I feel that having like a digital health coach will not be able to give you the human touch. I suppose, the feeling of talking to someone who will be able to give you a response that's specifically to what you want and not like what they all say - general idea. … So I think it'd be very hard for you to really feel that this person is able to relate to your feelings and like provide you with that emotional support that you're looking for.” |
| *FGD3, Student 004* |
| **Perceived technological limitations of CA** |
| “Yeah and, of course, the concern would be, what if the chatbot, AI, algorithm or whatever isn’t calibrated properly and it sends out the wrong response at the wrong time? It may trigger, perhaps even something as extreme as like a suicidal ideation and stuff like that” |
| *Mental health stakeholder 019, Associate Psychologist* |
| So I think it’ll be tricky if it's something to appeal to a mass audience, because you want to catch as many people as possible… Personally, I would prefer something that is more specific, because then it feels like the chatbot like understands what I'm going through and is able to speak to my specific situation, but I feel like that might not work if it needs to be suitable for a large audience. |
| *Mental health stakeholder 017, Mental Health First Aider* |
| Theme 6: Cultural considerations |
| **Local narrative** |
| “it’s pretty interesting, when each time they go and see a counsellor, it's always pertaining to two main issues, one is academic performance, and then another career related, it's always a performance indicator kind of stuff right.” |
| *Mental health stakeholder 005, Counsellor* |
| **Global context** |
| “And again, this is my own naivete within Singapore and the huge and amazing cultural mix that we have here, there may well need to be considerations made to the needs of the various groups and I couldn't speak on behalf of any of them beyond my own, but that may change the way the bot would want to interact with them and the types of things they might offer as suggestions and support.” |
| *Mental health stakeholder 006, Faculty Member* |
| **Language consideration** |
| “The more relatable the chatbot seems, the more effective it is likely to be. I'm reminded of a telegram bot, I think, that was made by a local and let me see… I believe the telegram what was programmed so that it will respond in a very Singlish manner, so you could ask the bot, what time the bus was coming, and then it will respond like, you know, like a bus uncle and that sort in Singlish” |
| *Mental health stakeholder 019, Associate Psychologist* |
| “If let's say that [the mHealth intervention] is for people with mental health disorders, then I think if let's say the phrases can be, [endorsed by] people who actually went through mental health disorders themselves. … let’s say they can be shared with people who actually went through mental health [disorders]. The kind of like statements that they don’t like to hear, or statements that really helped them for feeling like they're being listened to. I think that would be very useful also” |
| *Mental health stakeholder 017, Mental Health First Aider* |
